# Profiling miRNA in Systemic Lupus Erythematosus Patients Adhering to a Mediterranean Diet: An Interventional Pilot Study

**DOI:** 10.3390/jcm15052077

**Published:** 2026-03-09

**Authors:** Rocío Gil-Gutiérrez, Irene Medina-Martínez, María José Membrive-Jiménez, Antonio M. Caballero-Mateos, Francisco Javier de la Hera-Fernández, Nuria Navarrete-Navarrete, María Correa-Rodríguez, Blanca Rueda-Medina

**Affiliations:** 1 Department of Nursing, Faculty of Health Sciences, University of Granada, 18071 Granada, Spain; rogilgu@ugr.es (R.G.-G.); macoro@ugr.es (M.C.-R.); blarume@ugr.es (B.R.-M.); 2 CTS-436 Group, University of Granada, 18016 Granada, Spain; 3 Instituto de Investigación Biosanitaria ibs. GRANADA, 18012 Granada, Spain; javijanjan@gmail.com (F.J.d.l.H.-F.); nurianavarreten@ugr.es (N.N.-N.); 4 Sport and Health University Research Institute (iMUDS), University of Granada, 18007 Granada, Spain; 5 Faculty of Health Sicences, University of Granada (UGR), 18071 Granada, Spain; irene.medina.mrtnz@gmail.com; 6 Ceuta Faculty of Health Sciences, University of Granada, Cortadura del Valle s/n, 51001 Ceuta, Granada, Spain; mjmembrive@ugr.es; 7 Department of Internal Medicine, Gastroenterology Section, Santa Ana Hospital, Andalusian Health Service, Av. Enrique Martín Cuevas, s/n, 18600 Motril, Granada, Spain; 8 Systemic Autoimmune Diseases Unit, Hospital Universitario San Cecilio, 18016 Granada, Spain; 9 Systemic Autoimmune Diseases Unit, Hospital Universitario Virgen de las Nieves, 18014 Granada, Spain

**Keywords:** autoimmune diseases, epigenetics, extra virgin olive oil, mediterranean diet, miRNA, systemic lupus erythematosus

## Abstract

**Background/Objectives**: To analyze possible epigenetic changes (miRNA) in systemic lupus erythematosus (SLE) patients on a Mediterranean diet (MD) supplemented with extra virgin olive oil (EVOO). **Methods**: Fifteen SLE patients with medium/high MD adherence were randomized into an intervention group (IG) (daily supplementation of 40 mL of EVOO for 24 weeks) or to a control group (CG). miRNA profiles from blood peripheral cells were analyzed pre-/post-intervention using next-generation sequencing. Differential expression analysis was performed by DESeq2 in R to determine changes in the log2FC. Functional enrichment analysis was performed using GeneCodis 4. **Results**: EVOO supplementation resulted in changes in the expression of 16 miRNAs in the IG. Compared to the CG, two miRNAs showed upregulation (miR-451a, miR-1307-5p) while five showed downregulation (miR-193b-50, miR-134-5p, miR1287-5p, miR-124-3p, miR-654-3p). miR-124-3p, which has been proposed to be an SLE biomarker, showed the lowest relative expression after EVOO supplementation (L2FC −3.36; *p_unadj_* = 0.025), whereas miR-1307-5p (L2FC 1.115 *p_unadj_* = 0.02) and miR-451a (L2FC 0.77 *p_unadj_* = 0.036) showed the highest relative abundance. The functional enrichment analysis showed that Th1 and Th2 cell differentiation and the complement/coagulation cascades were among the top ten most significantly enriched pathways. **Conclusions**: Our data suggest that MD supplementation with EVOO leads to changes in the profile of miRNAs in SLE patients, potentially impacting disease pathogenesis. Further research is needed to validate these preliminary findings and the mechanisms by which EVOO modifies miRNA expression in the context of this disease.

## 1. Introduction

The Mediterranean diet (MD) is one of the best-known and studied dietary patterns worldwide. It is a plant-based dietary pattern characterized by predominant use of Extra Virgin Olive Oil (EVOO), high intake of whole plant foods, moderate consumption of dairy products and wine, and low meat intake that has been directly linked to a decrease in the risk of the onset of chronic conditions, decreased mortality and healthy aging promotion. The mechanisms underlying these beneficial effects include [1,2] a reduction in blood lipids; modulation of inflammatory, immune, and oxidative stress markers; improvement in insulin sensitivity; enhancement of endothelial and antithrombotic function; prevention of platelet aggregation; modulation of intestinal microbiome and epigenetic processes; influence on hormonal and growth factor activity relevant to cancer development; interference with nutrient-sensing mechanisms; and support of metabolic health. Previous studies have suggested that these beneficial effects could be mainly attributed to extra virgin olive oil (EVOO) as its main component. EVOO is a natural rich source of polyphenols, monounsaturated fatty acids (MUFAs) and other bioactive compounds [3] with confirmed anti-inflammatory, antimicrobial and antioxidant properties [4].

Given its anti-inflammatory and immunomodulatory potential, EVOO has also been studied in the context of autoimmune diseases, including systemic lupus erythematosus (SLE), although evidence is still scarce. Recent research suggests that components of the MD [5] and especially EVOO [6,7], contribute to modulating the immune activity and inflammatory pathways involved in SLE, potentially influencing disease progression and symptom severity. In murine models of pristane-induced SLE, EVOO-enriched diets attenuated kidney health and reduced inflammatory activity by activating antioxidant pathways and suppressing key pro-inflammatory signalling routes [8]. Similarly, supplementation with oleocanthal, a phenolic compound found in EVOO, helped preserve kidney function and vascular health [9]. In agreement with these findings, an in vitro study demonstrated that the phenolic fraction of EVOO reduced T-cell activation and inflammatory cytokine production in patients with SLE [10]. While these findings are promising, the specific molecular mechanisms through which EVOO exerts its effects in the context of SLE remain largely unknown. Among other mechanisms, there is a strong line of evidence that suggests that EVOO induces epigenetic changes in the human genome, including DNA methylation, histone modifications, and modulation of microRNA expression [11].

MicroRNAs (also known as miRNAs) are evolutionarily conserved small non-coding RNAs regulating gene expression at the post-transcriptional level and exerting a modulatory effect on cellular processes such as proliferation, metabolism, cellular growth, differentiation or apoptosis. They have been confirmed to be important regulators of homeostasis [12] and alteration of their expression is associated with the pathological mechanisms underlying diseases, including metabolic diseases, cancer or autoimmune conditions. Indeed, it has been demonstrated that there is an altered pattern of miRNA expression in many autoimmune diseases [13].

Interestingly, epigenetic changes, including changes at the miRNA level, have also been associated with SLE susceptibility and/or clinical course [14]. SLE patients exhibit decreased expression of miR-145, miR-142, and miR-125a in T cells, thus contributing to immune hyperactivation. Additionally, increased expression of miR-524-5p promotes the production of IFN-γ in activated T cells, thereby intensifying the inflammatory state [15]. Similarly, elevated miR-7, miR-21, miR-22 and miR-30a levels, which leads to an inappropriate activation and increased autoantibody production, have been observed in B cells from lupus patients [15]. In addition, reduced miR-361-5p, miR-128-3p, and miR-181-2-3p levels have been detected in plasmacytoid dendritic cells (pDCs) and are associated with increased production of type 1 interferon, a key factor in the pathogenesis of SLE [16]. Notably, miR-21 and miR-155 are overexpressed in peripheral blood mononuclear cells (PBMCs) in patients with SLE, especially those with lupus nephritis [17]. Furthermore, miRNAs such as miR-146a or miR-361-5p have been correlated with clinical course, with miR-146a appearing to play a crucial role in immune alterations and organ damage in SLE, thereby modulating both inflammation and disease-related complications. miR-146a expression is significantly elevated in SLE patients with high disease activity, and a positive correlation has been observed between the levels of miR-146a and IL-6, a proinflammatory cytokine [18]. Similarly, the level of miR-361-5p in cells has been related to disease activity (SLEDAI ≥ 4) [19].

Among the various environmental factors known to modulate epigenetic responses, nutrition has emerged as a particularly compelling area of interest. Specifically, evidence for the epigenetic effects of EVOO consumption and its potential positive impact in human health has been found in inflammatory conditions such as cancer, cardiovascular diseases or diabetes, as well as for some autoimmune conditions, mainly rheumatoid arthritis (RA), inflammatory bowel disease, or psoriasis [20]. However, most studies have been carried out in vitro or in animal models and have provided different results due to the use of different cellular systems, EVOO compounds and selection of candidate miRNAs. Thus, it has been difficult to establish a causal relationship and the exact physiological consequences of the epigenetic modification induced by EVOO consumption and its phenolic compounds [11]. Additionally, the specific role of EVOO as a potential modulator of the miRNA profile in SLE, and thus its impact at the epigenetic level in this condition, has not yet been investigated. In light of the above, the present pilot study aims to investigate the effect of dietary EVOO supplementation on the expression profile of miRNAs in a SLE cohort.

## 2. Materials and Methods

### 2.1. Design

A two-arm, prospective, randomized controlled interventional pilot study over a 24-week period was conducted in the context of the clinical trial “EFINUTRILES” NCT05261529 (ClinicalTrials.gov).

### 2.2. Study Population

A total of 23 women diagnosed with SLE from the Systemic Autoimmune Diseases Unit at the Hospital Universitario Clínico San Cecilio and Hospital Universitario Virgen de las Nieves (Granada, Spain) were included in the study. Diagnosis of SLE was established following the revised American College of Rheumatology (ACR), SLICC or ACR/EULAR criteria of 2019. Inclusion criteria were (1) SLE diagnosis at least one year ago, (2) stable SLEDAI-2K, with no treatment modifications, in the previous 3 months, and (3) medium (8–11 points) to high (12–14 points) adherence to an MD as measured by the 14-point MD adherence scale from the PREDIMED study [21]. The following exclusion criteria were established: (1) terminal stages of SLE, (2) serum creatinine levels ≥ 1.5 mg/dL, (3) type 1 diabetes mellitus, (4) infection, trauma or surgery in the six months prior to the intervention, (5) SLICC > 5, (6) pregnancy or breastfeeding, (7) diagnosis of other autoimmune/inflammatory diseases, (8) psychiatric or cognitive disorders hindering the ability to understand the research team’s indications.

### 2.3. Intervention

To reinforce MD adherence, and as part of baseline assessment and prior allocation, participants were reminded of the basic principles of MD by a nutritionist through a one-hour face-to-face session to ensure adequate levels of adherence to MD throughout the intervention. Subsequently, randomization and allocation were performed using the Oxford Minimization and Randomization computer-supported centralized OxMar system (Oxford Minimization and Randomization^®^, O’Callaghan CA, Oxford, UK), as it ensures allocation concealment and minimizes selection bias through a minimization algorithm, thereby complying with the Consolidated Standards of Reporting Trials (CONSORT) recommendations. A 1:1 distribution to intervention group (IG) or control group (CG) was applied.

Patients assigned to the IG added a daily supplementation of 40 mL of EVOO in a single dose, preferably at breakfast, for 24 weeks, whereas participants in the CG maintained their habitual dietary habits. Specific drinking cups were provided to ensure the correct dosage. EVOO was provided by the DCOOP (S.Coop.And) agri-food corporation. Laboratory tests were performed to ensure the stability of the EVOO composition during the intervention. Participants were given specific instructions not to process or cook the specific EVOO supplementation. No side effects related to the intervention were reported by participants in the intervention group (IG). Only one patient experienced mild heartburn when taking the supplement on an empty stomach; she was advised to take it with breakfast, and the side effect resolved. Patients were reminded weekly to take their supplementation, and any doubts or questions were resolved using group broadcast messages via instant messaging applications.

Blinding was applied at the investigator level. The researcher conducting the assessments was unaware of the participant’s allocation, and participants were instructed not to disclose their group during follow-up visits. However, full blinding was not feasible because the nature of the intervention (EVOO consumption or non-intervention) was evident to participants.

### 2.4. miRNA Sequencing

Blood samples were obtained from study participants pre- and post-intervention and collected in Tempus^®^ tubes (Applied Biosystems TM, Foster City, CA, USA). RNA was extracted using the MagMax for Stabilized Blood Tubes RNA Isolation Kit (ThermoFisher, Waltham, MA, USA) and a DynaMag-2 stand (Life Technologies, Carlsbad, CA, USA) at the Andalusian Public Health System Biobank (Granada, Spain). miRNA expression profile quantification was performed by creating a small RNA library using the QIAseq miRNA Library kit (QIAGEN, Hilden, Germany). Using Next-Generation Sequencing (NGS) technology, mass sequencing of miRNAs was performed on the NextSeq 500 (Illumina, San Diego, CA, USA) with a 72 base pairs (pb) single-read sequencing that yielded 4–8 million reads per sample.

Briefly, raw sequencing data obtained in the data binary base call (BCL) sequence file were demultiplexed using bcl2fastq conversion software (Illumina, v2.20.0.422, Illumina Inc., San Diego, CA, USA) to obtain Fastq files. Quality control (QC) checks on raw sequence data analysis were performed with FastQC software (v.0.12.1, Babraham Bioinformatics, Cambridge, UK) and reports were visualized together using Multiqc (v1.25.2, Seqera Labs, Barcelona, Spain). For unique molecular identifiers (UMIs) and adapter trimming, a custom tool written in Python (v1.0.2, GENYO, Granada, Spain) was employed. Quality trimming of reads was performed using Cutadapt (v4.4 Marcel Martin, TU Dortmunt University, Dortmund, Germany) with a phred threshold of 10. Reads shorter than 10 bp or longer than 40 bp were discarded. Next, reads were aligned against the GRCh38 human reference genome and the count matrix for human miRNAs was obtained using a mix of feature counts and a custom tool developed in Python using the miRbase database as reference. Differential expression analysis (DEA) was performed using DESeq2 in R (v 4.4.1, R Foundation for Statistical Computing, Vienna, Austria) and a series of R tools, such as ggplot2 (v3.5.1, Posit PBC, Boston, MA, USA) and pheatmap (v1.0.12, Raivo Kolde, Univerdity of Tartu, Estonia), were employed for the plotting. Significant differential expression of miRNAs was considered as |log2FC| > 0.6. For these differences, unadjusted (*p_unadj_*) and adjusted *p* values (*p_adj_*) were calculated for multiple testing using the Benjamini–Hochberg correction.

Functional enrichment analysis was performed using the bioinformatic tool GeneCodis 4 (https://genecodis.genyo.es, accessed on 3 July 2024) to investigate the molecular mechanisms and biological processes associated with the potential differentially expressed miRNAs.

### 2.5. miRNA Differential Expression and Pathway Analysis

miRNA expression analyses were performed at the “Centro Pfizer—Universidad de Granada—Junta de Andalucía de Genómica e Investigación Oncológica” (GENYO) Bioinformatics unit. The entire pipeline is publicly available under a GNU GPL 3.0 License at https://github.com/bioinfo-genyo/miRNA_pipe, accessed on 15 April 2024.

#### 2.5.1. Preprocessing and Quantification of miRNA-Seq Data

Sequencing data in BCL format were demultiplexed using Illumina’s bcl2fastq software (Illumina, v2.20.0.422) with no index mismatches to generate FASTQ files. Initial quality control of raw FASTQ files was performed using FastQC and summarized with MultiQC to assess read quality and guide trimming parameters. Deduplication and adapter trimming were then carried out using a Python-based strategy customized for the Qiagen QIAseq miRNA Library Kit. Quality trimming with parameters appropriate for small-RNA sequencing (10 bp minimum read length, 40 bp maximum read length and a minimum quality [PHRED] score of 10) was applied using cutadapt.

Robust and computationally efficient quantification of miRNA abundance was achieved implementing a two-step quantification strategy integrating direct sequence assignment (“precounting”) and feature-based counting using the featureCounts tool. In the initial quantification step, the custom Python method was used to directly assign sequencing reads to mature miRNA sequences from the mature miRBase database (release 22.1, filtered for the species and biotype of interest). Reads with exact sequence matches were assigned to their corresponding miRNAs and counted, while unmatched reads were separated into a different FASTQ file for further analysis. As a second step, reads not assigned through direct matching were aligned to the GRCh38 reference genome using Bowtie with parameters optimized for small-RNA reads, and converted into sorted, indexed BAM files. These files were then processed with featureCounts to quantify reads mapping to RNAcentral (release 24) miRNA genomic features (GRCh38), using strand specificity and allowing overlaps.

To generate the final quantification matrix, miRNA counts from direct sequence assignment and feature-based counting were merged for each sample to yield the total abundance for each miRNA.

#### 2.5.2. Differential Expression Analysis

A quantification matrix was used for downstream expression analyses. First, quality control was included to remove miRNAs with variance below the first percentile across all samples to reduce noise. Count matrix and sample annotation were combined using DESeqDataSetFromMatrix. A differential expression analysis was then conducted using the DESeq2 package in R. Default parameters were used in DESeq2 for normalization, employing median-of-ratios size-factor estimation and negative binomial dispersion modeling.

Significant differential expression of miRNAs was considered as |log2FC| > 0.6. For these differences, unadjusted (*p_unadj_*) and adjusted *p* values (*p_adj_*) were calculated for multiple testing using the Benjamini–Hochberg correction. Differentially expressed miRNAs were classified as “UP” or “DOWN”-regulated based on fold-change direction and significance threshold. Volcano plots were constructed with ggplot2 v3.5.1 and ggrepel v0.9.5 for dynamic labeling.

#### 2.5.3. Pathway Analysis

Functional enrichment analysis to investigate the molecular mechanisms and bio-logical processes associated with the potential differentially expressed miRNAs was performed using the bioinformatic tool Gene-Codis 4 (https://genecodis.genyo.es, accessed on 3 July 2024). The differentially expressed miRNAs were introduced as input in the Gene-Codis web tool and the KEEG Pathways selected as annotation database. Default Gene-Codis 4 parameters were used to obtain a network of enrichment pathways for the miRNAs analysed.

### 2.6. Ethics

The study protocol was approved by the Biomedical Research Ethics Committee of Granada, Spain (2099-N-21) according to the Helsinki Declaration for biomedical research. Informed consent was obtained from all patients prior to participation. The study received prior approval from the regional ethics committee before any patient contact. The recruitment period extended from November 2021 to May 2022. One potential participant was approached on 3 November 2021, before trial registration on ClinicalTrials.gov (8 February 2022), but withdrew prior to formal enrollment and was excluded from all analyses. All participants included in the final dataset were recruited after trial registration, in full compliance with ICMJE requirements.

## 3. Results

**Figure 1:**
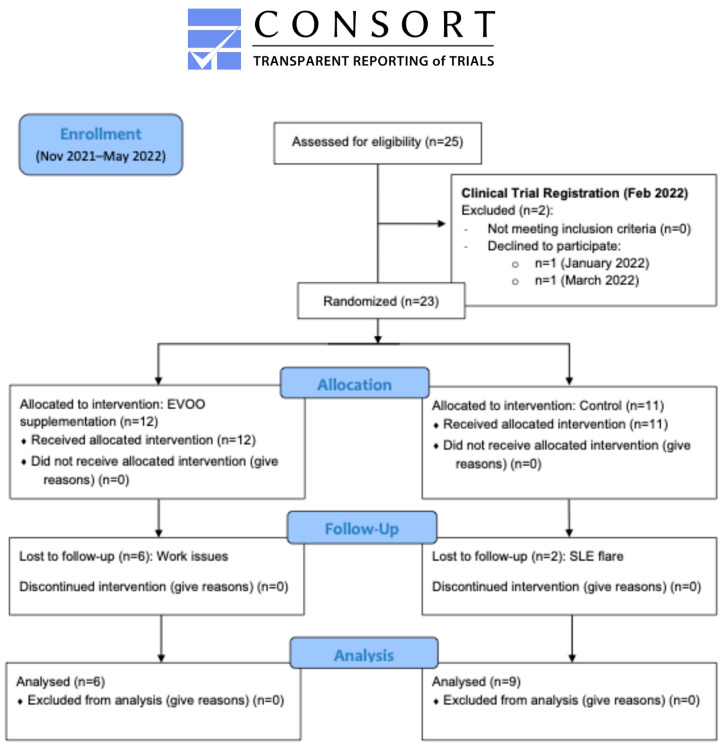
Study flow diagram according to CONSORT guidelines.

Of the 23 eligible women enrolled after registration, 12 (52.17%) were randomly assigned to the intervention group (extra virgin olive oil supplementation), and 11 (47.83%) to the control group. From this initial sample, six participants (50%) in the intervention group discontinued (three due to work reasons, two for personal issues, and one for family caregiving), and two participants (18.18%) in the control group withdrew (one due to an SLE flare and another due to a non-SLE health issue). These details are summarized in the CONSORT flow diagram (Figure 1).

G* Power software (1996 version, GPOWER, Bonn, Germany) was used to perform a post hoc power analysis in the context of a two-group comparison using a two-sided independent samples *t*-test. Considering that six and nine participants completed the intervention for each group (IG and CG, respectively), and assuming a significance level of 0.05 and an effect size based on conventional benchmarks (Cohen’s d = 0.8) [22], the statistical power achieved for this interventional pilot study was estimated to be 0.29.

The average age for the total sample was 49.80 ± 9.59 years, with an MD adherence level corresponding to medium adherence (9.13 ± 1.12 points) according to the 14-point DM adherence scale. No significant differences were observed between groups in terms of sociodemographic variables, in any of the SLE-related clinical severity or damage indexes, or in the prescribed pharmacological treatments (*p* > 0.05 in all cases) (Table 1). Mean SLEDAI-2K and SLICC values were 3.80 ± 3.60 and 0.60 ± 0.82, respectively, which remained stable throughout the entire study period (SLDAI-2K and SLICC post intervention values were 3.92 ± 3.98 and 0.42 ± 0.64, respectively, *p* > 0.05 in both cases).

**Figure 2:**
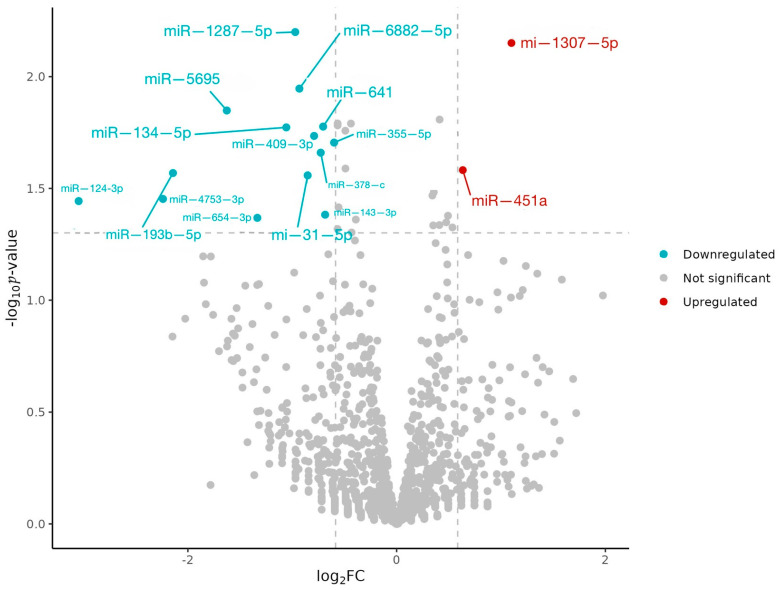
Vulcan plot of the miRNAs differentially expressed post-intervention in the IG. Statistical significance in this volcano plot is defined using unadjusted *p*-values (as depicted by the horizontal reference line), and the interpretation of differentially expressed miRNAs should be made accordingly.

A total of 1560 miRNAs were identified in the analyzed samples. In the intervention group (IG), 34 miRNAs showed differential expression after daily EVOO supplementation. Of these, 16 exhibited statistically significant differential expression based on unadjusted *p*-values (|log_2_FC| > 0.6), with two upregulated (miR-1307-5p and miR-451a) and fourteen downregulated (Figure 2). Unfortunately, none of these miRNAs remained significant after multiple-testing correction due to the limited statistical power of this exploratory study (Table 2). No statistically significant changes in these miRNAs—either unadjusted or adjusted—were observed in the control group (Table 2).

The miRNA with the highest relative abundance was miR-1307-5p (L2FC 1.10, *p_unadj_* = 0.007, *p_adj_* non-significant; ns), whereas the miRNA that showed the lowest relative expression after EVOO supplementation was miR-124-3p (L2FC −3.046, *p_unadj_* = 0.036, *p_adj_* ns) (Table 2).

Table 3 shows a comparison of the 16 miRNAs differentially expressed between the IG and CG after EVOO supplementation. As can be seen, the differential expression for the two upregulated miRNAs was statistically significant when comparing IG with CG although the significance disappears when correcting for multiple testing (miR-1307-5p L2FC 1.115 *p_unadj_* = 0.02 and miR-451a L2FC 0.77 *p_unadj_* = 0.036, *p_adj_* ns). For the 14 downregulated miRNAs, the comparison between IG and CG revealed that the differential expression was statistically significant without adjustment for multiple testing only for miR-193b-5p, miR-134-5p, miR-1287-5p, miR-124-3p and miR-654-3p. Again, the miRNA that showed the lowest relative abundance was miR-124-3p (L2FC −3.36; *p_unadj_* = 0.025, *p_adj_* ns) followed by miR-193b-5p (L2FC −3.05; *p_unadj_* 0.00, *p_adj_* ns).

**Figure 3:**
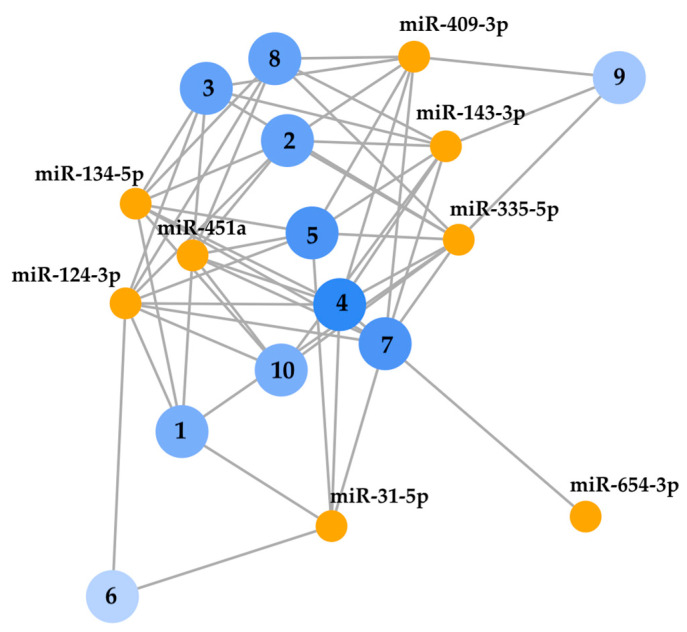
Top ten networks of differentially expressed miRNAs. Numbers represent networks with significant enrichment (*p* < 0.01): 1. Neuroactive ligand-receptor interaction; 2. Leishmaniasis; 3. Th1 and Th2 cell differentiation; 4. ErbB signalling pathway; 5. Platelet activation; 6. Non-homologous end-joining; 7. Progesterone-mediated oocyte maturation; 8. GnRH secretion; 9. Complement and coagulation cascades; 10. Aldosterone-regulated sodium reabsorption. The blue intensity goes in concordance with the number of genes found. Nodes that share genes are close together.

We used the KEGG tool in GeneCodis 4 to analyze, in a descriptive exploratory context, the pathway enrichment of the differentially expressed miRNAs after EVOO supplementation. This showed several networks with significant enrichment (Supplementary Material Document S1), with Th1 and Th2 cell differentiation and the complement and coagulation cascades, which are involved in the pathogenesis of SLE, being among the top ten most significantly enriched (Figure 3).

## 4. Discussion

In the present interventional pilot study, we have investigated the effect of a nutritional intervention based on supplementation of the MD with 40 mL EVOO daily for 24 weeks in SLE patients on the epigenetic changes at the miRNA expression levels for the first time. We observed differences in the abundance of 16 miRNAs post-intervention, with two miRNAs upregulated and 14 downregulated. These preliminary findings should be considered with caution, and require confirmation in future, adequately powered studies.

miR-1307-5p showed the highest abundance post-intervention. While this miRNA has been linked to several positive [23] and negative [24] cancer-related outcomes, to date it has not been associated with any of the mechanisms involved in the pathological process of SLE or any other autoimmune condition. Likewise, although to a lesser extent, we found increases in miR-451a expression post-intervention. In animal models, an increase in miRNA-451a expression has been observed in immune organs, while downregulation of this miRNA leads to a reduction in autoreactive T lymphocytes and proinflammatory cytokine levels. These studies have shown that miRNA-451a targets the INF regulatory factor (IRF8) gene, amongst others [25,26]. Conversely, a study in patients revealed that reduced levels of miRNA-451a in serum exosomes correlate with disease activity and renal damage [27]. Thus, further studies are required to fully dilucidated the exact role of miR-451a in SLE.

Focusing on the 14 miRNAs whose expression levels decreased following the intervention, we observed that only miR-124-3p, miR-1287-5p, miR-134-5p, miR-5695, miR-335-5p, and miR-31-5p have been associated with specific biological functions related to SLE or other autoimmune conditions.

First miR-124-3p, which presents the lowest relative expression in IG compared to CG. Interestingly, miR-124-3p has already been proposed to be an SLE biomarker as its levels have been found to be significantly upregulated and independently associated with SLE remission rate. Blood plasma levels of this miRNA have been found to be positively and statistically significantly correlated with levels of erythrocyte sedimentation rate, C3 and C4 complements, and anti-C1q antibodies [28].

We also observed a decreased expression of miR-1287-5p, miR-134-5p and miR-5695 after EVOO supplementation. These miRNAs have been linked to inflammatory and/or autoimmune processes. Thus, increased miR-1287-5p levels are associated with pro-inflammatory effects [29]. Notably, recent evidence in traumatic spinal cord injury reports that miR-1287-5p downregulation associates with clinical severity metrics and that experimental overexpression dampens inflammatory cytokines via direct targeting of MAP3K9, suggesting a potential anti-inflammatory role for lower miR-1287-5p in that context; taken together, these observations may indicate context-dependent effects of miR-1287-5p on inflammatory signalling [30]. Similarly, miR-134-5p and miR-5695 have been found in the miRNA panel characteristic of RA patients [31,32].

Continuing with the exploratory description of the intervention’s effects, we observed reductions in the expression of miR-335-5p and miR-31-5p. miR-335-5P has been associated with osteoarthritis, where its abnormal overexpression promotes chondrocyte apoptosis and contributes to cartilage degradation via HMG-box transcription factor 1 (HBP1) [33], whereas an in vitro study demonstrated that miR-31-5p interacts with solute carrier family 15 member 4 (SLC15A4), enhancing its expression and activating the TLR7 signaling pathway in pDCs of patients with lupus [34,35]. Because TLR7 signalling and dysregulated plasmacytoid dendritic cell responses are central to SLE pathogenesis, changes in these miRNAs could be of potential relevance for disease-related inflammatory pathways. However, in the context of this pilot study, these findings should be regarded as preliminary signals. They may nonetheless help outline candidate pathways and generate hypotheses to be examined in future, adequately powered studies.

With regard to the pathway enrichment of differentially expressed miRNAs post-intervention provided by KEGG, we should highlight Th1 and Th2 cell differentiation, platelet activation and the complement and coagulation cascades, since all of them are pathways involved in SLE pathogenesis implicated in immune response dysregulation, release of inflammatory mediators [36], complement activation, inflammation, or tissue damage associated with this disease [37]. Overall, the KEGG output aligns with pathways central to SLE course, suggesting a plausible biological context for the observed profiles; nonetheless, with few miRNAs and no multiplicity adjustment obtained, these should be read strictly as descriptive hypotheses.

This interventional pilot study has limitations that should be considered when interpreting the results. The limited sample size and low statistical power represent a major constraint beyond the pilot nature of the study, substantially limiting the ability to detect clinically meaningful differences and increasing the risk of Type II error. This limitation is partly explained by the complexity of the intervention design, which involved a six-month intervention period that could interfere with participants’ usual routines, particularly in situations such as travel or eating outside the home, where adherence to the protocol (e.g., carrying and using the assigned oil) may be challenging. Although only three study visits were required, participants belonged to a population frequently approached for multiple research protocols, leading to a phenomenon that can be defined as “research fatigue. Recruiting individuals with chronic conditions, such as autoimmune diseases, poses additional challenges as their willingness to participate is often shaped by prior experiences with intensive research demands and a perception that investigator priorities may not fully align with their own expectations or daily realities. These considerations differ markedly from those of healthy volunteers, whose motivations and perceived burdens are typically less complex [38]. Furthermore, the high dropout rate—particularly within the intervention group—introduces a potential source of selection bias. Participants who remained in the study may differ systematically from those who withdrew (e.g., in motivation, adherence capacity, symptom stability, or perceived burden of the protocol), potentially influencing the characteristics of the final cohort and the resulting estimates of intervention effects. These differential attrition patterns further restrict the generalizability of the findings, as the results may largely reflect a subset of individuals able or willing to maintain adherence to a six-month dietary intervention rather than the broader population living with autoimmune conditions. Additionally, the inclusion of advanced molecular analyses, such as miRNA profiling, required high-cost procedures and substantial funding, which further restricted the feasibility of expanding the sample size. These factors posed significant challenges for recruitment and retention, ultimately limiting the generalizability of the findings.

Therefore, given the exploratory nature of this study, the findings should be regarded as hypothesis-generating rather than confirmatory, particularly in the context of adjusted *p*-values. Future studies should consider strategies to reduce participant burden and secure adequate resources to support larger, long-term intervention trials with larger and consistent results. However, the results appear to indicate a tentative trend aligned with the proposed hypothesis, although the limited sample size and unadjusted *p*-values warrant cautious interpretation of the observed effect sizes. Thus, validation in a larger study cohort, including experimental mechanistic validation, is warranted to confirm the relevance of these preliminary findings. In this same context, and given the pilot nature of the study, we intentionally required moderate–high baseline adherence to the Mediterranean diet to isolate the incremental effect of EVOO; however, future larger studies should evaluate whether different baseline adherence levels differentially influence intervention outcomes. In addition, the possibility that uncontrolled lifestyle factors during the intervention period (i.e., changes in rest, physical activity patterns) could have exerted an influence at the epigenetic level should be considered. However, this possibility seems remote as one of the strengths of this study lies in the inclusion of stable and well-balanced patient groups. There were no significant changes in the levels of adherence to the MD, medical treatment or clinical parameters among participants, which are the factors that could have most significantly influenced the expression levels of miRNAs. Furthermore, there were no changes in menopausal status or BMI, both of which are known to influence miRNA expression. This contextual consideration supports the robustness and interpretability of these exploratory findings.

## 5. Conclusions

In conclusion, these preliminary results provide novel scientific evidence for how a change in the usual diet (EVOO supplementation) could influence SLE patients at the epigenetic level by modulating the expression of different miRNAs. Together with previous studies on miRNA function, it seems that the up-regulated and down-regulated miRNAs could result in beneficial effects at the level of different regulatory mechanisms altered in the pathogenesis of SLE.

## Figures and Tables

**Table 1:** Baseline characteristics.

Outcome		Total (n = 15)	IG (n = 6)	CG (n = 9)	*p* Value ^C^
Sociodemographic characteristics					
Age (years) ^A^		49.80 ± 9.59	50.16 ± 5.74	40.55 ± 11.84	0.909
Menopause	No (n) ^B^	5 (33.3)	2 (33.3)	3 (33.3)	1.000
	Yes (n) ^B^	10 (66.7)	4 (66.7)	6 (66.7)	
Hemodynamic status	SBP (mmHg) ^A^	118.86 ± 10.74	117.33 ± 13.27	119.88 ± 9.42	0.668
	DBP (mmHg) ^A^	72.26 ± 6.16	70.66 ± 8.59	73.33 ± 5.22	0.465
	HR (bpm) ^A^	65.66 ± 10.58	64.00 ± 9.77	66.77 ± 11.53	0.636
BMI (kg/m^2^) ^A^		23.97 ± 5.32	26.21 ± 6.85	22.47 ± 3.73	0.192
MD adherence (points) ^A^		9.12 ± 1.12	9.33 ± 1.21	9.00 ± 1.11	0.593
Clinical Severity and Damage Measures					
Years since SLE diagnosis ^A^		19.93 ± 8.91	18.66 ± 7.58	20.77 ± 10.05	0.670
SLEDAI2-K (points) ^A^		3.80 ± 3.60	5.00 ± 3.57	3.00 ± 3.60	0.310
SLICC/ACR (points) ^A^		0.60 ± 0.82	0.66 ± 1.03	0.55 ± 0.72	0.810
Pharmacological treatment					
Corticoids ^B^		7 (46.7)	2 (33.3)	5 (55.6)	0.435
Azathioprine ^B^		1 (6.7)	1 (16.7)	0 (0)	0.363
Mycophenolate mofetil ^B^		0 (0)	0 (0)	0 (0)	-
Methotrexate		3 (20.0)	1 (16.7)	2 (22.2)	0.810
Other immunosuppressants		1 (6.7)	0 (0)	1 (11.1)	0.435
Statins		2 (13.3)	2 (33.3)	0 (0)	0.175
NSAIDs		5 (33.3)	2 (33.3)	3 (33.3)	1.000
Antihypertensive drugs		5 (33.3)	1 (16.7)	4 (44.4)	0.297
Rituximab		0 (0)	0 (0)	0 (0)	-
Belimumab		0 (0)	0 (0)	0 (0)	-
Antimalarials		12 (80.0)	6 (100)	6 (66.7)	0.081
Antiplatelet agents		3 (20.0)	0 (0)	3 (33.3)	0.081
Vitamin D supplements		12 (80.0)	5 (83.3)	7 (77.8)	0.810
Calcium supplements		3 (20.0)	1 (16.7)	2 (22.2)	0.810
Bisphosphonates		2 (13.3)	0 (0)	2 (22.2)	0.169

Data are expressed as ^A^ median and standard deviation (m ± SD) for continuous data or ^B^ number and frequency (n, %) for qualitative variables. ^C^ Student’s t/Mann–Whitney U (continuous), chi-squared/Fischer’s exact (categorical), based on distribution. Two-sided *p*-values were used for baseline comparisons, as no directional hypothesis was assumed. - indicates test not applicable due to zero variance. Abbreviations: BMI: body mass index; CG: control group; DBP: diastolic blood pressure; HR: heart rate; IG: intervention group; m: mean value; MD: Mediterranean diet; NSAIDs: non-steroidal anti-inflammatory drugs; SBP: systolic blood pressure; SD: standard deviation; SLEDAI2-K: Systemic Lupus Erythematosus Disease Activity Index 2000; SLICC/ACR: Systemic Lupus International Collaborating Clinics/American College of Rheumatology Damage Index for Systemic Lupus Erythematosus.

**Table 2:** miRNAs showing statistically significant differential expression after EVOO supplementation.

Intervention Group (n = 6)				
miRNA	L2FC median value	*p_unadj_*	*p_adj_* *	Expression change
miR-1287-5p	−0.971	0.006	1	down
miR-1307-5p	1.100	0.007	1	up
miR-6882-5p	−0.931	0.011	1	down
miR-5695	−1.627	0.014	1	down
miR-641	−0.704	0.016	1	down
miR-134-5p	−1057	0.016	1	down
miR-409-3p	−0.790	0.018	1	down
miR-335-5p	−0.599	0.019	1	down
miR-378c	−0.727	0.021	1	down
miR-451a	0.634	0.026	1	up
miR-193b-5p	−2.142	0.026	1	down
miR-31-5p	−0.852	0.027	1	down
miR-4753-3p	−2.238	0.035	1	down
miR-124-3p	−3.046	0.036	1	down
miR-143-3p	−0.685	0.041	1	down
miR-654-3p	−1334	0.042	1	down
Control Group (n = 9)				
miRNA	L2FC median value	*p_unadj_*	*p_adj_* *	Expression change
miR-1287-5p	0.066	0.824	0.901	no
miR-1307-5p	0.054	0.896	0.939	no
miR-6882-5p	−0.639	0.039	0.176	no
miR-5695	−0.759	0.118	1	no
miR-641	−0.728	0.004	0.065	no
miR-134-5p	0.360	0.262	0.490	no
miR-409-3p	−0.140	0.623	0.795	no
miR-335-5p	−0.431	0.050	0.203	no
miR-378c	−0.571	0.036	0.164	no
miR-451a	−0.028	0.905	0.943	no
miR-193b-5p	0.970	0.087	1	no
miR-31-5p	−0.340	0.304	0.531	no
miR-4753-3p	−1.853	0.038	1	no
miR-124-3p	0.347	0.747	1	no
miR-143-3p	−0.131	0.648	0.799	no
miR-654-3p	0.216	0.642	0.799	no

* Adjusted *p* value: non-significant in all cases. Abbreviations: miRNA: microRNA; L2FC: log2 Fold Change. Expression change: up (upregulation), down (downregulation), no (no change in expression).

**Table 3:** Comparison of the miRNAs differentially expressed between intervention and control groups.

miRNA	L2FC Median Value	*p_unadj_*	*p_adj_* *	Expression Change
miR-1287-5p	−0.926	0.016	0.090	down
miR-1307-5p	1.115	0.020	0.090	up
miR-6882-5p	−0.187	0.630	0.727	no
miR-5695	−0.796	0.281	0.401	no
miR-641	0.103	0.720	0.772	no
miR-134-5p	−1.354	0.004	0.061	down
miR-409-3p	−0.579	0.098	0.196	no
miR-335-5p	−0.085	0.701	0.772	no
miR-378c	−0.162	0.620	0.727	no
miR-451a	0.773	0.035	0.107	up
miR-193b-5p	−3.050	0.002	0.061	down
miR-31-5p	−0.430	0.329	0.449	no
miR-4753-3p	−0.320	0.799	0.827	no
miR-124-3p	−3.357	0.025	0.092	down
miR-143-3p	−0.521	0.180	0.281	no
miR-654-3p	−1.461	0.040	0.110	down

* Adjusted *p* value: non-significant in all cases. Abbreviations: miRNA: microRNA; L2FC: Log Fold Change. Expression change: up (upregulation), down (downregulation), no (no change in expression).

## Data Availability

Data supporting the findings obtained are available on appropriate request from the corresponding author and are not available publicly for ethical reasons.

## Supplementary Materials

The following supporting information can be downloaded at: https://www.mdpi.com/article/10.3390/jcm15052077/s1, Document S1: Analysis of biological pathways influenced by miRNAs in which significant expression changes were identified after intervention.

## Abbreviations

The following abbreviations are used in this manuscript:

ACR	American College of Rheumatology
BCL	Binary Base Call
BMI	Body Mass Index
CG	Control Group
CD8+ T cells	Cytotoxic T lymphocytes
DBP	Diastolic Blood Pressure
DEA	Differential Expression Analysis
DNA	Deoxyribonucleic acid
EULAR	European Alliance of Associations for Rheumatology
EVOO	Extra Virgin Olive Oil
GNU GPL	General Public License
HDAC	Histone deacetylases
HR	Heart Rate
IFN-γ	Interferon Gamma
IRF8	Interferon regulatory factor 8
IG	Intervention Group
kg	Kilograms
LDL-c	Low-Density Lipoprotein Cholesterol
log2FC	Log Fold Change
m	meters
MD	Mediterranean Diet
miRNA	Micro ribonucleic acid
microRNA	Micro ribonucleic acid
MUFAs	Monounsaturated Fatty Acids
NGS	Next-Generation Sequencing
NF-kB	Nuclear factor kappa B
NSAIDs	Non-steroidal anti-inflammatory drugs
ns	Non-significant
PBMCs	Peripheral Blood Mononuclear Cells
*p_adj_*	Adjusted *p*-value
pDCs	Plasmacytoid Dendritic Cells
*p_unadj_*	Non-adjusted *p*-value
QC	Quality Control
RA	Rheumatoid Arthritis
RNA	Ribonucleic Acid
SBP	Systolic Blood Pressure
SLC15A4	Solute carrier family 15 member 4 human gene
SLE	Systemic Lupus Erythematosus
SLEDAI	Systemic Lupus Erythematosus Disease Activity Index
SLEDAI-2K	Systemic Lupus Erythematosus Disease Activity Index 2000
SLICC	Systemic Lupus International Collaborating Clinics
TLR7	Toll-like receptor 7
UMIs	Unique Molecular Identifiers
